# Occupational Lung Disease Causing Allergic Bronchopulmonary Aspergillosis: A Case Report

**DOI:** 10.7759/cureus.66252

**Published:** 2024-08-06

**Authors:** E. Ramya Shree, Deepan Kumar T, C. H. Naga Sekhar, Krishnaswamy Madhavan, J. S. Kumar

**Affiliations:** 1 Department of General Medicine, SRM Medical College Hospital and Research Centre, Kattankulathur, IND

**Keywords:** allergic bronchopulmonary aspergillosis (abpa), cystic fibrosis, neutrophils, pulmonary tuberculosis, aspergillus fumigatus

## Abstract

*Aspergillus fumigatus* can induce allergic bronchopulmonary aspergillosis (ABPA), an immunological hypersensitivity reaction that frequently exacerbates the symptoms of cystic fibrosis and asthma patients. Due to persistent symptoms, a considerable percentage of patients with ABPA in India, a country where tuberculosis is widespread, are initially misdiagnosed as having pulmonary tuberculosis. We present a case of ABPA in a male industry worker, who was diagnosed after one year of having symptoms and has successfully recovered since.

## Introduction

A hypersensitivity reaction to inhaled *Aspergillus* spores, allergic bronchopulmonary aspergillosis (ABPA) is usually linked to a history of asthma or cystic fibrosis. ABPA is a pulmonary immune-mediated illness caused by *Aspergillus*, a fungus that colonizes the respiratory systems of people suffering from cystic fibrosis and asthma [1]. Some uncommon appearances may make diagnosis challenging, particularly if there is no history of asthma. ABPA affects around 2.5 million individuals globally, with those employed in agriculture, construction, and other industries being the most affected [2].

In cases of ABPA, wheezing is frequently observed. Invasive aspergillosis has an almost 100% fatality rate if treatment is not received [3]. A complete diagnostic workup is required in situations of suspected invasive aspergillosis; however, treatment should start as soon as feasible to lower morbidity and death. Exacerbations of ABPA are usually treated with itraconazole to reduce the fungal burden and corticosteroids to regulate the immunological response. For patients with moderate functional changes, the prognosis for ABPA is favorable [4]. If the diagnosis is postponed, many people need to take steroids for an extended duration. Lung fibrosis and steroid resistance can arise as a result of delayed diagnosis.

## Case presentation

A 50-year-old gentleman presented with breathlessness for six months, gradual in onset, chronic, and severely progressive, with cough and mucoid expectoration, associated with high-grade fever with loss of appetite in the last 10 days. He had lost 5 kg in the last two months. The patient had a similar episode one month ago, which was treated with doxycycline and Deriphyllin, and he was discharged. The patient had no history of asthma or cystic fibrosis.

**Figure 1:**
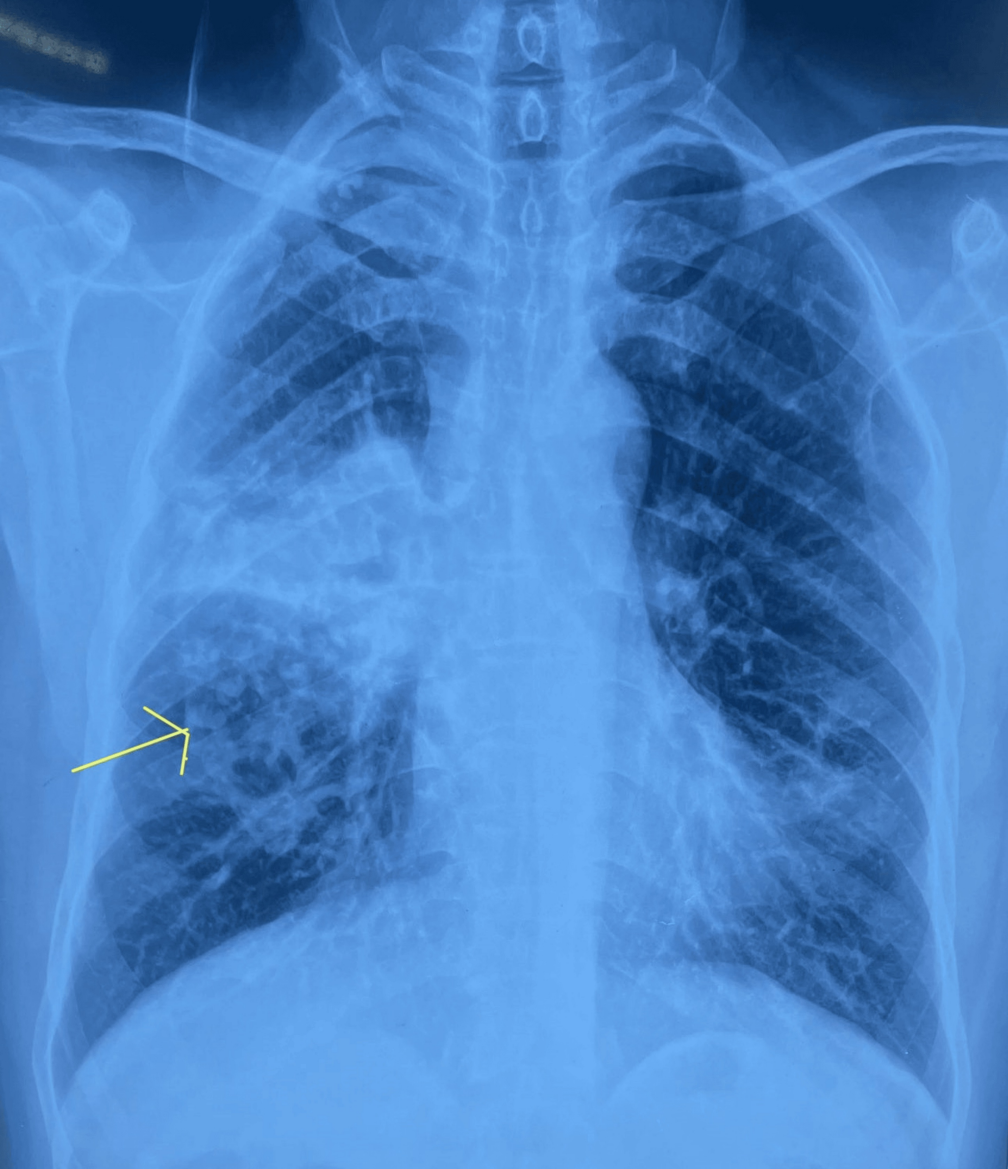
Chest X-ray posteroanterior view showing heterogenous, interstitial opacities in the middle zone.

The patient’s occupational history revealed crucial clues about the case. He worked in the plastic manufacturing industry for more than 25 years, where he was exposed to continuous inhalation of fumes. The chronic inhalation of polyvinyl chloride (PVC) fumes may have resulted in a chronic bronchitis-like picture, which was undiagnosed for a long time. Due to recurrent respiratory illness, he had to quit his job and started working in box making and motor company. He denied the use of tobacco or alcohol. He had a polypectomy done for bilateral nasal polyps 15 years ago. Pre-anesthetic chest X-ray assessment for an inguinal hernial repair showed the findings illustrated in Figure 1.

**Figure 2:**
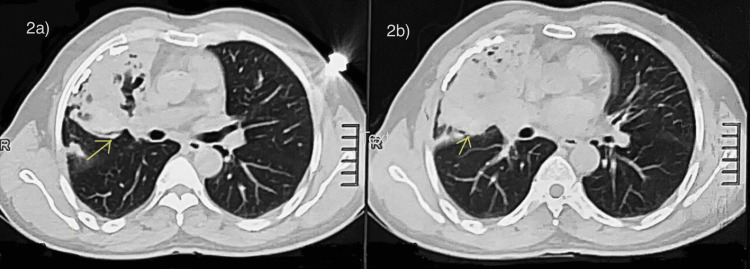
(a, b) CT of the chest showing minimal pneumonitis with fibrotic bronchiectatic strands on the lateral segment of the right middle lobe.

He underwent sputum acid-fast bacilli and chest CT for further analysis. CT of the chest showed sub-pleural minimal pneumonitis, fibrotic strands with bronchiectasis, and a heterogenous subpleural nodular lesion in the lateral segment of the right middle lobe (Figure 2). The workup performed five months ago ruled out for tuberculosis or malignancy. A repeat CT of the thorax after three months showed cystic bronchiectasis with surrounding consolidation with volume loss in the right anterior segment of the upper lobe. Bronchoscopy with a biopsy was done. The first biopsy showed respiratory mucosa with underlying stroma showing mild chronic inflammatory cells infiltrative with focal areas of hemorrhage, with no evidence of malignancy, and the second biopsy was suggestive of a chronic inflammatory lesion, but no evidence of granuloma. Pulmonary function tests were performed, with forced expiratory volume in the first second/forced vital capacity showing moderate obstruction (69.6%).

**Figure 3:**
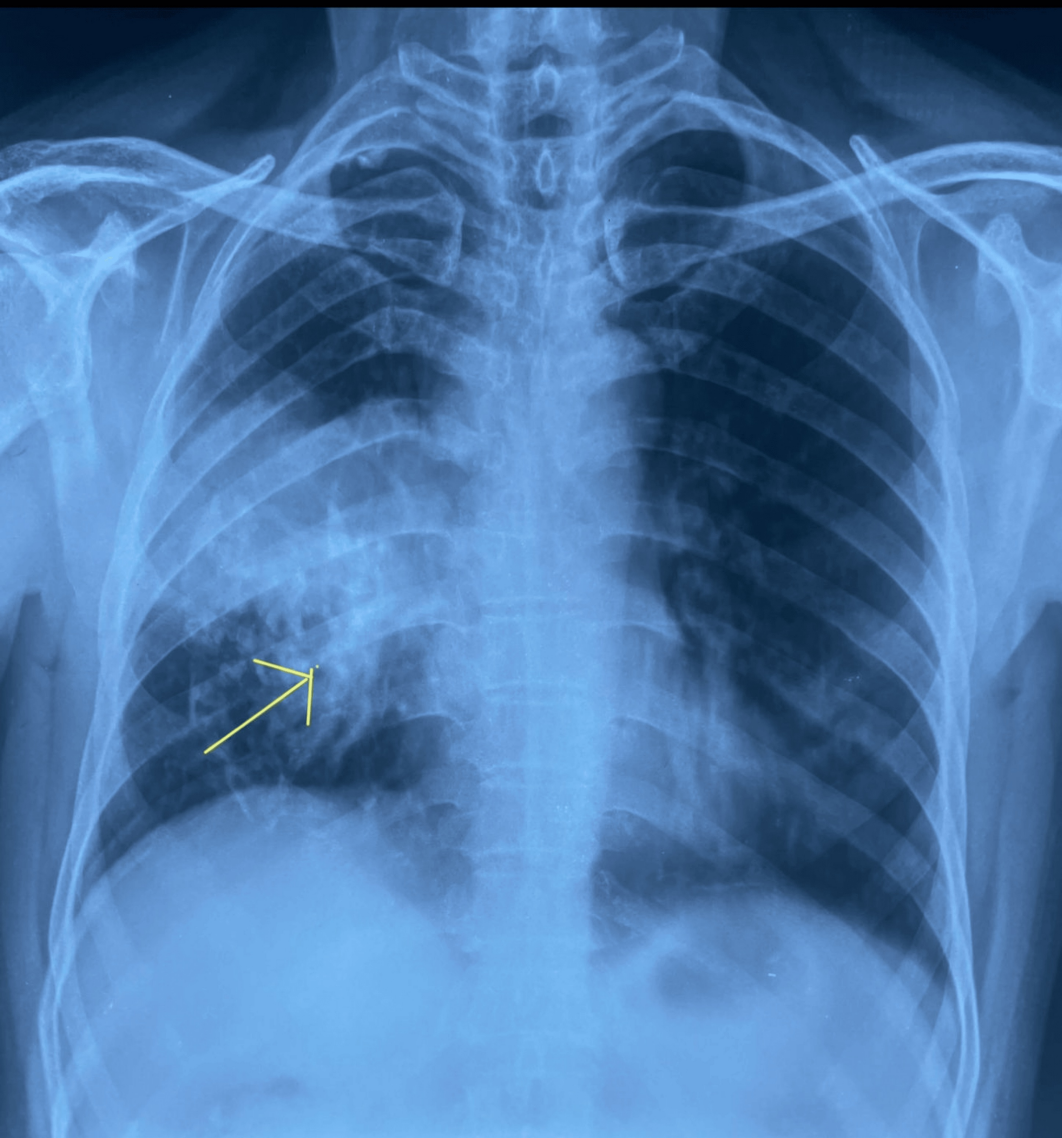
Chest X-ray posteroanterior view showing the increased right middle zone to lower zone haziness/consolidation compared to the previous chest X-ray (taken six months earlier).

Presently, on clinical examination, the patient showed absent air entry, a dull note on percussion, and decreased vocal fremitus and vocal resonance in the right upper and middle lobe. Blood investigations revealed an elevated white blood cell (WBC) count with increased neutrophil and eosinophil. Other parameters were normal. Peripheral smear showed moderate normocytic, normochromic anemia with neutrophilic leucocytosis. Compared to previous chest X-rays, the current X-ray showed increased consolidation in the middle and lower zones (Figure 3). A CT-guided biopsy showed a chronic inflammatory lesion with interstitial fibrosis. A pulmonology opinion was sought, and it was advised to start chest physiotherapy and postural drainage and to rule out ABPA or post-infection sequelae. On further review, they advised bronchoalveolar lavage (BAL), galactomannan, and pan-fungal assay. BAL showed the presence of mucus plugs, few bronchioles, dense interstitial fibrosis, collagenization, and moderate individual to small clusters of inflammatory cells with lymphocytes, plasma cells, and pigment-laden macrophages. Raised absolute eosinophil count (>1,000) and raised IgE levels pointed to allergic reactions in the patient. IgE-specific antibody for ABPA was elevated (1,687 ng/mL).

**Figure 4:**
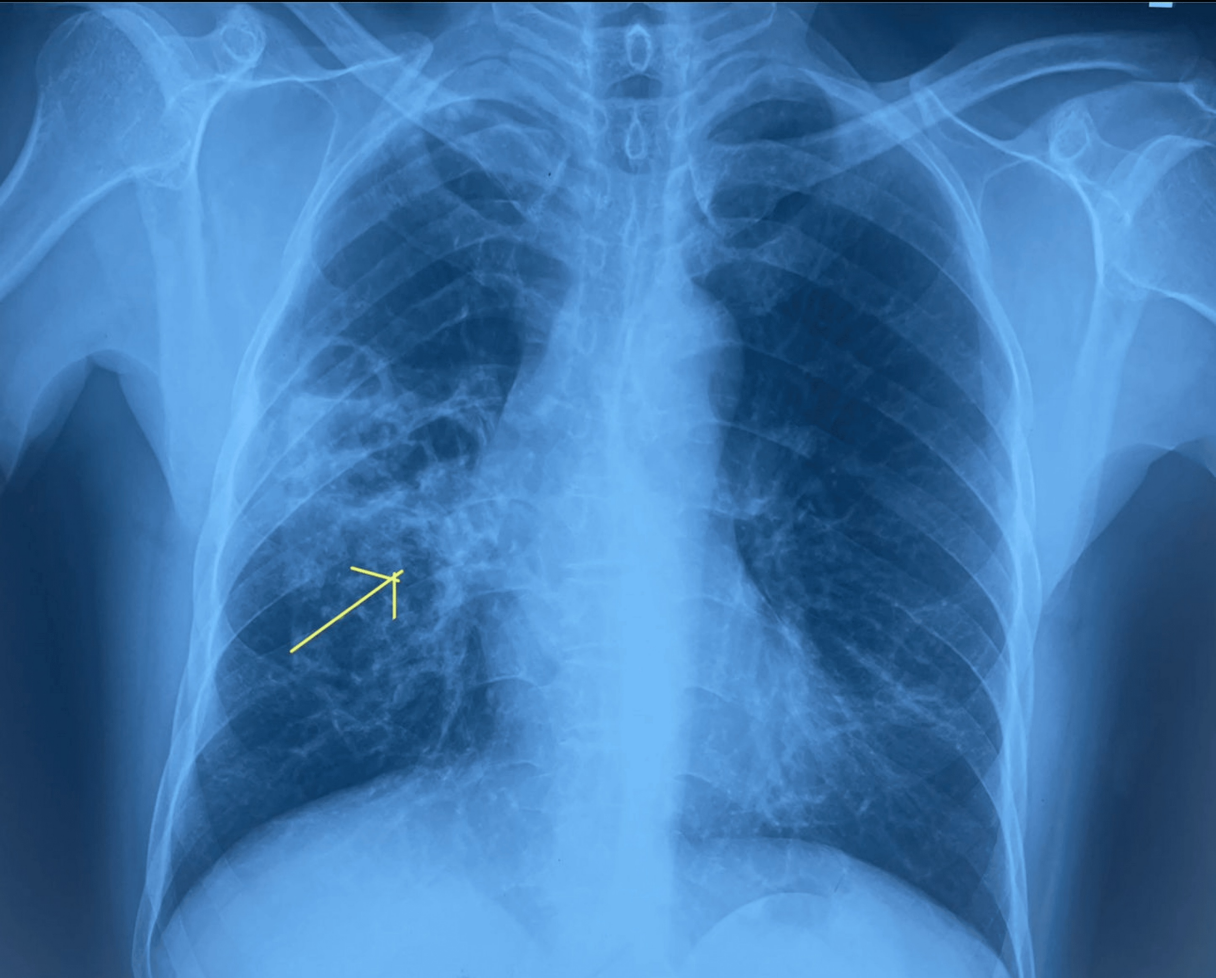
Chest X-ray after treatment showing improvement with reduction in opacities.

This patient had all types of respiratory pathology such as restrictive lung disease, bronchiectasis, asthma, and occupational lung disease due to plastic fumes, and was positive for allergic aspergillosis. The patient was suspected to have Young syndrome because of a history of polyps and male infertility but was later excluded due to lack of evidence. Due to elevated WBC count, febrile status, and chest X-ray showing right middle lobe opacity, the patient was diagnosed with community-acquired pneumonia and was initiated on parenteral, oral, and bronchodilators. After the treatment, the patient’s respiratory auscultation findings improved from decreased air entry to bronchial breathing, and his vocal fremitus and vocal resonance increased. Figure 4 shows the patient’s chest X-ray after treatment.

## Discussion

*Aspergillus fumigatus* is the causative organism in ABPA, which was also the cause in our case. Clinically, ABPA patients exhibit cough, dyspnea, anorexia, low-grade fever, weight loss, and wheeze and/or crepitations on auscultation, in addition to severe asthmatic symptoms. Diagnosing this illness remains challenging, especially in cases of atypical clinical presentations like our patient’s, who had difficulty breathing, but no history of asthma symptoms or diagnosis [5]. Indeed, relatively asymptomatic ABPA presents in one-third of patients with managed asthma. The diagnosis is made during regular testing, as was the case in our instance when the disease was discovered during a routine check-up while preparing for hernia surgery. Similar to our case, an ABPA case was reported in 2021 in a lady who had no known history of asthma [6].

During the early course of the disease, chest X-ray reveals a normal study, but as the disease progresses, it shows transient patterns such as “Tramline shadows” and “finger in glove-like opacities,” indicative of edema and bronchial wall thickening. Bilateral infiltrations with a “ring shadow” on an X-ray suggest bronchiectasis. The above-mentioned chest X-ray findings appear in most ABPA cases but were not seen in our patient [7].

A study from India observed that out of 126 patients with ABPA who presented to a chest clinic in 2006, 59 were first misdiagnosed as pulmonary tuberculosis and were treated with antitubercular treatment (ATT) [8]. Comparatively, a 2009 retrospective analysis found that ATT was initially recommended for pulmonary tuberculosis (91% of patients with ABPA). When the patient’s findings satisfy the requirements outlined in 2013 by the International Society for Human and Animal Mycology group, an ABPA diagnosis is verified. Two of the three criteria-positive serum precipitins/*Aspergillus fumigatus* IgG, an eosinophil blood count of more than 500 cells/L, and CT of the chest showing features of ABPA, such as mucus impaction, tree-in-bud pattern, and centri lobular nodules, are sufficient for the diagnosis of ABPA, provided total IgE level is more than 1,000 IU/mL [9]. Our diagnosis was supported by our patient’s total eosinophil count of greater than 500 cells/L and increased total IgE levels of ABPA. Various treatments have demonstrated efficacy in the management of ABPA. Glucocorticoids are the first course of treatment for APBA cases. A randomized trial demonstrated that high-dose and medium-dose regimens are equally effective against ABPA, with the medium-dose regimen having fewer side effects [10].

Antifungal medication may be added in glucocorticoid-dependent individuals. Omalizumab, an anti-IgE medication with a steroid-sparing action, may be a useful alternative for ABPA in patients with steroid resistance or an additional therapeutic option. Albuterol was also administered via a nebulizer machine in our case [11]. Chest radiography and total serum IgE levels should be checked every two months until remission. A patient is considered to be in remission when they have not experienced any flare-ups for at least six months following the cessation of all treatments. A minimum of 25% decrease in total IgE levels is required in response to therapy, along with improvements in clinical and radiological outcomes [12]. However, a favorable prognosis is achieved when the disease is detected early and prompt treatment is initiated. Respiratory failure and irreversible lung fibrosis develop in patients when no adequate treatment is started.

## Conclusions

ABPA is frequently misdiagnosed as tuberculosis or pneumonia, among other pulmonary conditions. This is a case of an occupational lung disease due to chronic inhalation of PVC plastic fumes, which damaged the lung causing ABPA, with a picture of mixed obstructive and restrictive pathology. Hence, this case demonstrates the need for comprehensive therapy techniques in complex lung disorders and emphasizes the significance of investigating different diagnoses in individuals exhibiting overlapping symptoms.
